# Individual Differences in Sensorimotor Adaptation Are Conserved Over Time and Across Force-Field Tasks

**DOI:** 10.3389/fnhum.2021.692181

**Published:** 2021-11-30

**Authors:** Robert T. Moore, Tyler Cluff

**Affiliations:** ^1^Cumming School of Medicine, University of Calgary, Calgary, AB, Canada; ^2^Hotchkiss Brain Institute, University of Calgary, Calgary, AB, Canada; ^3^Faculty of Kinesiology, University of Calgary, Calgary, AB, Canada

**Keywords:** motor learning, adaptation, savings, individual differences, motor memory, force-field adaptation, visuomotor adaptation

## Abstract

Sensorimotor adaptation enables the nervous system to modify actions for different conditions and environments. Many studies have investigated factors that influence adaptation at the group level. There is growing recognition that individuals vary in their ability to adapt motor skills and that a better understanding of individual differences in adaptation may inform how motor skills are taught and rehabilitated. Here we examined individual differences in the adaptation of upper-limb reaching movements. We quantified the extent to which participants adapted their movements to a velocity-dependent force field during an initial session, at 24 h, and again 1-week later. Participants (*n* = 28) displayed savings, which was expressed as greater initial adaptation when re-exposed to the force field. Individual differences in adaptation across various stages of the experiment displayed weak-strong reliability, such that individuals who adapted to a greater extent in the initial session tended to do so when re-exposed to the force field. Our second experiment investigated if individual differences in adaptation are also present when participants adapt to different force fields or a force field and visuomotor rotation. Separate groups of participants adapted to position- and velocity-dependent force fields (*Experiment 2a*; *n* = 20) or a velocity-dependent force field and visuomotor rotation in a single session (*Experiment 2b*; *n* = 20). Participants who adapted to a greater extent to velocity-dependent forces tended to show a greater extent of adaptation when exposed to position-dependent forces. In contrast, correlations were weak between various stages of adaptation to the force-field and visuomotor rotation. Collectively, our study reveals individual differences in adaptation that are reliable across repeated exposure to the same force field and present when adapting to different force fields.

## Introduction

Motor learning encompasses a range of neural and behavioral processes that play a role in producing skilled actions. These processes can occur on short timescales, like a golfer adjusting their aim to accommodate gusting winds, to longer timescales that modify actions throughout growth, development, and aging (Shadmehr et al., 2010). Sensorimotor adaptation produces short-term changes in actions that enable the nervous system to manipulate objects or move in environments with varying physical properties.

Adaptation is often studied by examining how the nervous system modifies motor actions when exposed to a visual rotation or force field that systematically disrupts the accuracy of movement (Shadmehr et al., 2010; Krakauer et al., 2019). The nervous system adapts over the course of tens to hundreds of trials and reduces the effect of the disturbance until movements become relatively accurate again. Healthy adults tend to display savings, expressed as faster adaptation when re-exposed to a visuomotor rotation or force field (Morehead et al., 2015; Coltman et al., 2019; Nguyen et al., 2019). Factors including sensory feedback (Coltman and Gribble, 2020; Crevecoeur et al., 2020a, b; Mathew et al., 2020, 2021), reinforcement (Huang et al., 2011; Leow et al., 2013; Galea et al., 2015), and damage to the nervous system influence the average rate and amount of adaptation (Takahashi and Reinkensmeyer, 2003; Smith and Shadmehr, 2005; Rabe et al., 2009; Donchin et al., 2011; Mutha et al., 2011) as well as the amount of savings (Leow et al., 2012, 2013).

It is well-established that adaptation varies across individuals, and there is growing consensus that this variation may be biologically meaningful (Seidler et al., 2015; Seidler and Carson, 2017). Individual differences in visuomotor adaptation are associated with variation in spatial working memory (Anguera et al., 2010), proprioception (Tsay et al., 2021), brain activity and structure (Della-Maggiore et al., 2009, 2017; Anguera et al., 2010; Donchin et al., 2011; Ruitenberg et al., 2018), and may reflect differences in how individuals update their representation of the visuomotor rotation (Oh and Schweighofer, 2019). Importantly, individual differences display moderate-strong reliability with repeated exposure to the same visuomotor rotation, suggesting variation across individuals reflects the unique ways in which participants adapt their movements (Stark-Inbar et al., 2017; Wilterson and Taylor, 2021).

There may be some differences in the way individuals adapt to visuomotor rotations and force fields. Factors including age and proprioception seem to have different influences on visuomotor and force-field adaptation (Kitchen and Miall, 2020). These findings compliment evidence that different brain regions are engaged in visuomotor and force-field adaptation (Rabe et al., 2009; Donchin et al., 2011). Research on force-field adaptation has primarily focused on group averages. There is evidence that force-field adaptation measured within a single session is associated with individual differences in brain activity (Vahdat et al., 2011, 2014). It remains unclear if individual differences in adaptation are reliable with repeated exposure to the same force field.

Here we examined how healthy young adults adapt their reaching movements when repeatedly exposed to a force field. The participants encountered a velocity-dependent force field in an initial session, 24 h later, and again 1-week after the session at 24-h (*Experiment 1*; 8 days total). We examined the amount participants adapted when they initially encountered the forces, after extended practice, and when the forces were removed unexpectedly after adaptation. Based on evidence in visuomotor adaptation (Stark-Inbar et al., 2017), we hypothesized that participants would display reliable individual differences in force-field adaptation. Consistent with our hypothesis, the results revealed reliable individual differences in force-field adaptation that persisted for a week without practice.

Next, we questioned if individual differences are evident when participants adapt to different force fields or a force field and visuomotor rotation. Separate groups of participants adapted their reaching movements to position- and velocity-dependent force fields (*Experiment 2a*) or a force field and visuomotor rotation (*Experiment 2b*). Adapting to position- and velocity-dependent forces may rely on common neural and behavioral processes, which can create interference and impair performance when the forces are applied in opposing directions (Bays et al., 2005; Sing et al., 2009). In contrast, force-field and visuomotor adaptation may rely on separate neural structures and behavioral processes (Rabe et al., 2009; Donchin et al., 2011). Thus, we hypothesized that individual differences would correlate when participants adapted to different force fields, but such correlations would be weak or absent when adapting to a force field and visuomotor rotation. Consistent with this hypothesis, individuals displayed moderate-strong correlations in the amount they adapted their movements and reduced errors imposed by the position- and velocity-dependent force fields. In contrast, correlations were weak between the amount that participants adapted their movements and minimized errors produced by the force field and visuomotor rotation. Collectively, the results highlight individual differences when participants adapt their reaching movements to different force fields.

## Materials and Methods

### Participants

A total of 68 naïve participants were recruited from the University of Calgary and surrounding community [36 male; mean age = 23.41 (*SEM* = 0.43) years]. Participants reported no history of neurological or musculoskeletal disorders and had normal or corrected vision. Seven participants were left-handed based on self-report. The study protocol was approved by the Conjoint Health Research Ethics Board at the University of Calgary. Participants provided written informed consent before the experiments and were monetarily compensated for their time.

### Experimental Design

Participants performed reaching movements while seated with their arms supported in a robotic exoskeleton device (Kinarm, Kingston, ON, Canada). Visual targets and hand feedback were projected into the plane of the participant’s arm using an LCD monitor and semi-silvered mirror. Direct vision of the arm and hand were occluded by a physical barrier.

Participants reached to a single goal target with their dominant arm. Each trial began with a central start target displayed on the screen. The start target was positioned so that participants began each trial with 20° shoulder flexion relative to the frontal plane and 110° elbow flexion relative to the upper arm (external angle; 0° indicates full extension). Participants initiated the trial by moving a small feedback cursor (1.0 cm diameter white circle) into the start target and maintaining this position for a brief period (500 ± 200 ms uniformly distributed). The feedback cursor was aligned to the tip of the index finger unless otherwise specified (see *Experiment 2b*). The goal target then appeared 15 cm directly in front of the start target. Participants were instructed to move to the goal target within 500 ms of leaving the start target. Trial pacing was self-initiated. We did not impose any constraints or instruct participants to limit their reaction times. Participants were required to remain in the goal target for 750 ms to complete the trial. The task design is consistent with studies that impose both timing and accuracy demands in force-field adaptation (Nguyen et al., 2019; Avraham and Nisky, 2020; Crevecoeur et al., 2020a; Mathew et al., 2021). Following the hold period, we provided explicit timing feedback at the end of every trial. The goal target remained green and “Good Timing” was displayed on the screen when the participant met the timing demands and stabilized in the goal target. The goal target turned blue and “Speed Up” was displayed on the screen if the participant did not complete the movement and hold period within the allotted time window (1,250 ms). The goal target was then extinguished, and the start target reappeared on the screen to cue the participant to begin the next trial.

#### Experiment 1: Individual Differences in Adaptation With Repeated Exposure to the Same Force Field

Many studies have examined how participants adapt their reaching movements when they re-encounter the same force field (Caithness et al., 2004; Joiner and Smith, 2008; Coltman et al., 2019). On average, healthy participants display savings, or an improvement in initial adaptation, when re-exposed to the same force field within minutes, hours, days, or weeks of initial exposure (Krakauer et al., 1999; Caithness et al., 2004; Joiner and Smith, 2008; Coltman et al., 2019; Nguyen et al., 2019; Mathew et al., 2021). Here we examined individual differences in adaptation when participants were re-exposed to the same force field over three sessions.

**FIGURE 1:**
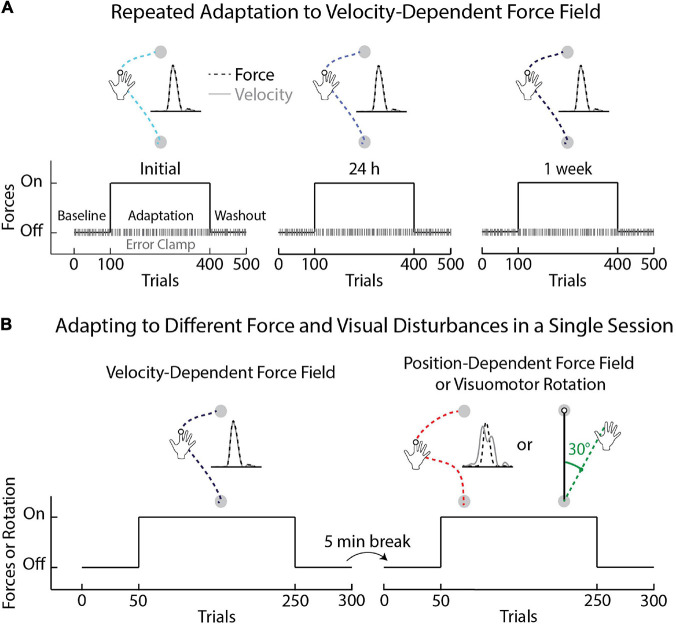
Schematic of experimental protocols. **(A)** Task protocol and time course of *Experiment 1*. Participants began the first session (Initial) by performing unloaded reaching movements (Baseline phase), followed by movements with velocity-dependent forces (Adaptation phase), followed by unloaded reaching movements (Washout phase). Error-clamp trials were interleaved throughout the experiment (20% of trials in each phase). The same protocol was repeated the following day (24 h) and again 1 week after the 24-h session (1 week). **(B)** Task protocols for *Experiments 2a* and *2b*. In *Experiment 2a* participants adapted to velocity- and position-dependent force fields. The magnitude of the position-dependent force field was identical across trials and independent from the velocity of movement. Participants in *Experiment 2b* adapted to a velocity-dependent force field and a 30° visuomotor rotation. The order of the tasks was counterbalanced across participants. The direction of the force fields and visuomotor rotation was flipped for left-handed participants.

Participants [*n* = 28; mean age = 24.96 (*SEM* = 0.66) years; 12 female; 26 right-hand dominant] reached from a start target (1.3 cm diameter) to goal target (2.0 cm diameter). They adapted their reaching movements to a velocity-dependent force field in three sessions—an initial session (Initial), a session 24 h later (24 h), and again 1 week after the session at 24 h (1 week; Figure 1A). The forces were orthogonal to the main direction of the reach and required participants to activate the extensors of the arm to resist the forces (Scheidt et al., 2000; Michel et al., 2018; Avraham and Nisky, 2020; Crevecoeur et al., 2020a; Mathew et al., 2021). The robot applied mechanical loads to the shoulder and/or elbow joints to create specific forces at the hand (leftward forces for right-handed participants). The force field produced 12 N of lateral force for every 1 m/s of forward hand velocity (*B*, Equation 1),


(1) $$F_{x}=-B\,\dot{y}$$

Participants were instructed to resist the forces while performing accurate movements that met the timing demands. Each block of trials consisted of eight movements in the force field and two movements in an error-clamp. The trials were presented in random order. During error-clamp trials, the robot created a mechanical channel that constrained hand motion to an approximately straight line. The channel was defined relative to the center of the start target and was created by applying elastic (600 N/m) and damping (15 Ns/m) forces that resisted hand motion orthogonal to the channel walls. Lateral hand motion was minimal in the clamp [mean = 0.17 (*SEM* = 0.01) mm], enabling measurements of the forces that participants exerted against the channel wall throughout the experiment (Smith et al., 2006; Joiner and Smith, 2008; Heald et al., 2018; Coltman et al., 2019; Nguyen et al., 2019). The channel stiffness and damping ramped up (400 ms sigmoidal ramp-to-hold profile) when the goal target appeared in the participant’s workspace (during the reaction time and early reach period). The size of the start target (1.3 cm diameter) combined with the ramp-up of the force channel helped to avoid participants receiving an explicit force cue, detecting the presence of the channel prior to movement, and any variability in force compensation it may have introduced between trials and participants.

The experiment consisted of 400 unconstrained movements and 100 pseudo-randomly interleaved error-clamp trials (Scheidt et al., 2000; Orban de Xivry et al., 2013; Kitchen and Miall, 2020). Participants first completed 100 unloaded movements to measure their nominal reaching patterns (80 null, 20 clamp trials). We then introduced the force field unexpectedly and at full strength. Participants performed a total of 300 trials to measure how they adapted to the forces (240 in force field, 60 clamp trials). The force field was then removed unexpectedly, and participants performed 100 trials to washout adaptation (80 null, 20 clamp trials). The experiment was completed in under 50 min in each session.

#### Experiment 2: Individual Differences in Adaptation Across Different Force Fields and Visuomotor Rotations

The first experiment investigated individual differences in adaptation across repeated exposure to the same velocity-dependent force field. We questioned if these differences in adaptation were specific to the velocity-dependent force field. Here, participants reached from a start target (2.0 cm diameter) to a goal target (2.0 cm diameter) while adapting to position- and velocity-dependent forces (*Experiment 2a*) or velocity-dependent forces and a visuomotor rotation (*Experiment 2b*). The force-field and visuomotor rotation tasks were encountered in a single session separated by washout, a 5-min break, and subsequent baseline trials in the absence of forces or visual rotations (Figure 1B).

In *Experiment 2a*, participants [*n* = 20; mean age = 22.15 (*SEM* = 0.53) years, 10 female; 18 right-hand dominant] adapted to the same velocity-dependent forces as *Experiment 1*. The amplitude and location of the peak forces differed from trial to trial due to variation in movement velocity. They also encountered position-dependent forces that peaked at the midpoint of the reach [force scaling (*a*) = 4.7; position of peak (*b*) = 7.5 cm; standard deviation (*c*) = 6.0 cm; Equation 2].


(2) $$F_{x}=a\,e^{-\frac{{\left(D\,i\,s\,t_{y}-b/y_{e\,n\,d}-y_{s\,t\,a\,r\,t}\right)}^{2}}{2\,c^{2}}}$$

The amplitude of the positional forces was consistent across trials and dependent on the position of the hand (*Dist*_*y*_) relative to the start and goal targets (*y*_*start*_ and *y*_*end*_). The amplitude and location of the peak forces differed across the velocity and position-dependent force fields and were more variable when countering the velocity-dependent forces (Supplementary Material). The amplitude and location of the peak forces did not correlate across tasks. Half of the participants started with the velocity-dependent forces, while the other half first adapted to the position-dependent forces. Participants performed 50 movements in baseline, 200 in adaptation, and 50 washout trials (Figure 1B). Each block consisted of 10 trials. After the washout phase, participants took a 5-min break before performing the second task. The experiment was completed in approximately 60 min.

In *Experiment 2b*, a separate group of participants [*n* = 20; mean age = 22.50 (*SEM* = 0.84) years; 10 female; 17 right-hand dominant] interacted with the same velocity-dependent forces as *Experiments 1* and *2a*. They also adapted to a visuomotor rotation (Figure 1B) that altered the relationship between the motion of their hand and the position of a real-time feedback cursor displayed in their workspace. Hand feedback was rotated 30° counter-clockwise relative to the center of the start target in the adaptation phase for right-handed participants. The rotation required that participants reach 30° to the right of the goal target (i.e., clockwise) to move their feedback cursor along a straight path from the start to goal target. Half of the participants started with the velocity-dependent force field. The other half started with the visuomotor rotation task. The overall protocol was the same as *Experiment 2a* (Figure 1B). The direction of the force fields and visuomotor rotation was flipped for left-handed participants in all experiments (Takahashi and Reinkensmeyer, 2003; Lefumat et al., 2016).

### Data Analysis

Angular motion of the shoulder and elbow joints was sampled at 1 kHz, stored, and digitally low-pass filtered prior to analysis (second-order, bidirectional Butterworth filter, 30 Hz effective cut-off). Hand coordinates were calculated from the measured joint angles at each time sample. We tracked adaptation in force field trials by measuring the peak lateral deviation (cm) between the participant’s hand path and a straight line connecting the center of the start target and goal target on each trial (Malfait et al., 2002; Mattar and Gribble, 2005; Anwar et al., 2011; Nasir et al., 2013; Kadota et al., 2014; Heald et al., 2018). We compared adaptation across sessions by quantifying the average peak lateral deviations in *Early Adaptation* (first three blocks of the adaptation phase) and *Late Adaptation* (last three blocks of the adaptation phase). We also quantified the average aftereffects, or peak lateral deviations, expressed in the first three blocks of the washout phase (*Washout*). Peak lateral deviations in each phase of adaptation were averaged across a similar number of trials as past studies (Stockinger et al., 2014; Avraham et al., 2020; Kitchen and Miall, 2020). We present analyses based on unnormalized peak lateral deviations (*Experiments 1* and *2*) but observed qualitatively similar results when we: (1) baseline reduced, (2) normalized peak lateral deviations in every trial to the largest deviation in the adaptation phase of each session (*Experiment 1*) or task (*Experiment 2*), and (3) used the lateral deviations measured at peak hand velocity.

In error-clamp trials (*Experiment 1*), we compared the instantaneous forces that participants exerted on the channel walls with the force required to resist the force field had it been applied (Smith et al., 2006; Joiner and Smith, 2008; McDougle et al., 2015; Heald et al., 2018; Coltman et al., 2019). We quantified adaptation using the slope of the linear regression between the ideal and participant-generated forces. The slope of the regression was multiplied by 100 to yield an estimate of force compensation (%) on each trial (Schween et al., 2020). We limited our analysis to the time period between movement onset and offset. Movement onset was defined as the time the participant’s instantaneous hand velocity first exceeded a threshold of 12.5% of the peak hand velocity for five consecutive samples. Movement offset was defined as the first time point the participant’s hand velocity fell below threshold for five consecutive samples. Qualitatively similar results were observed when the analyses were repeated using thresholds of 5% (Mattar and Gribble, 2005; Duff and Sainburg, 2007) and 10% peak hand velocity (Herzfeld et al., 2014a; Stockinger et al., 2014), as well as absolute thresholds of 1 cm/s (Heald et al., 2018) and 2 cm/s (Coltman et al., 2019). The error-clamp measures were averaged across the *Early Adaptation*, *Late Adaptation*, and *Washout* periods defined above.

Visuomotor adaptation (*Experiment 2b*) was quantified using the angular deviation of the cursor at 150 ms after the onset of each movement. This procedure allowed us to measure changes in planned hand paths while minimizing the influence of online visuomotor corrections (Telgen et al., 2014; Werner et al., 2014; Hayashi et al., 2016; Lefumat et al., 2016; Michel et al., 2018). Qualitatively similar results were obtained using peak lateral cursor deviations in the visuomotor rotation task (Supplementary Material).

### Statistical Analysis

Descriptive statistics are reported as the mean and standard error (SEM). We assessed the normality of our data using Lilliefors tests. Multivariate outliers were identified using Mahalanobis distances (Mahalanobis, 1936) combined with Wilks’ method (Wilks, 1963) and removed prior to analysis. In *Experiment 1*, we compared kinematic and clamp measures of adaptation across testing sessions (fixed-effects) using linear mixed-effects models. Participants were included as a random effect (Nguyen et al., 2019; Olivier et al., 2019). We performed ANOVA on the fixed effects of the model to test for differences across testing sessions. When the ANOVA revealed a significant difference across testing sessions, *post-hoc* comparisons between individual testing sessions were performed using *F*-tests (Meteyard and Davies, 2020). Savings was quantified by comparing kinematic and clamp-based measures in *Early Adaptation* across testing sessions (Leow et al., 2012; Coltman et al., 2019; Nguyen et al., 2019; Palidis et al., 2020). Measures of adaptation were compared across tasks (*Experiment 2*) using paired *t*-tests (two-tailed).

Correlation analysis was based on Pearson’s product moment correlation coefficient (*r*). We bootstrapped the correlations (*r*) by resampling 99,999 times with replacement (Racine and MacKinnon, 2007; Wilcox, 2009). We also calculated differences in the strength of the correlations (Δ*r*) and performed the same bootstrapping procedure to attain distributions of the differences in correlation coefficients. Bootstrapped *r* and Δ*r* values are presented throughout the text, tables, and figures. Correlations and differences in the strength of correlations were considered significant if <5% of the bootstrapped *r* or Δ*r* values crossed zero. Note the significance of the individual correlations had no bearing on the significance of differences in correlation strengths. The analyses were repeated with partial Pearson’s correlations that adjusted for individual differences in the inertia of the arm. The analyses were performed to rule out any influence of inertia when participants with different physical characteristics were exposed to the same force field or visuomotor rotation. The inertia of the arm (hand, forearm, and upper arm) was estimated based on standard anthropometric methods (Winter, 2009).

Bonferroni methods were used to correct for multiple comparisons and correlations on all kinematic and clamp-based measures of adaptation in *Experiment 1* (Bonferroni, 1936; 3 comparisons for each measure). Corrected *p*-values are reported throughout the text, tables, and figures. The results were considered significant if the corrected *p*-values were less than α = 0.05. Corrections were not applied in *Experiment 2* given that only one comparison and correlation was performed for each measure of adaptation. Data analyses were conducted using custom scripts written in MATLAB (MathWorks, Natick, MA).

## Results

### Experiment 1: Average Differences in Movement Kinematics Across Testing Sessions

**FIGURE 2:**
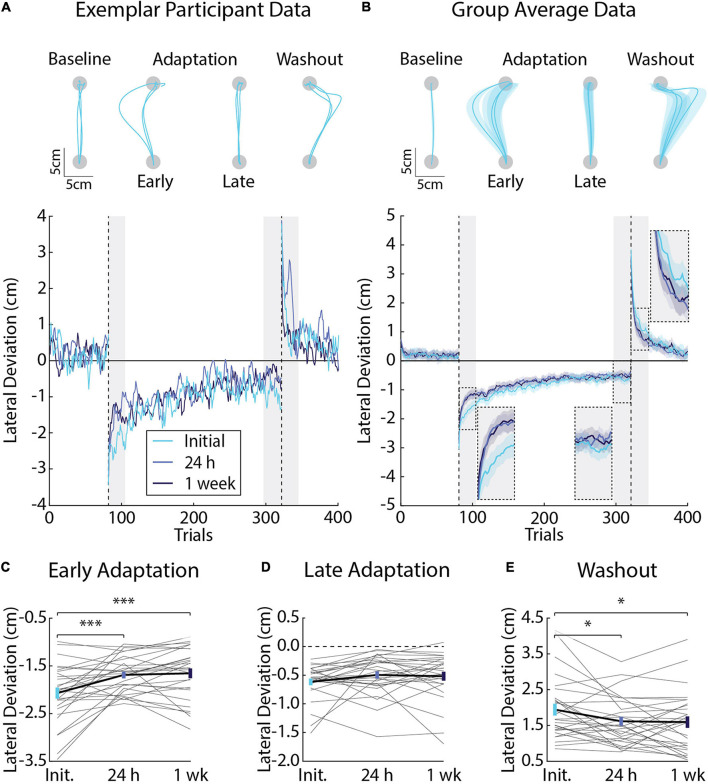
Differences in movement kinematics (peak lateral deviations) across testing sessions (*Experiment 1*). **(A)** Hand paths and adaptation profiles from an exemplar participant in *Experiment 1*. Hand paths were sampled at random for three trials in the baseline phase. The first and last three trials are shown for the adaptation phase. The first three trials are shown for the washout phase. Peak lateral deviations (cm) were measured on each trial and plotted for the exemplar participant. **(B)** Group average adaptation profiles (*n* = 28, 12 female). Lines represent the mean and shaded regions the SEM. Adaptation was measured in *Early Adaptation*, *Late Adaptation*, and *Washout* (shaded gray regions and inset panels). **(C)** Comparisons of the average peak lateral deviations measured in *Early Adaptation* in each session (Initial, 24 h, and 1 week). Gray lines indicate individual participant averages measured across sessions. Error-bars indicate mean and SEM during *Early Adaptation*. **(D)** Average peak lateral deviations in *Late Adaptation*. **(E)** Average peak lateral deviation in *Washout*. Data in panels **(D,E)** are represented in the same manner as panel **(C)**. *p < 0.05 and ***p < 0.001 after Bonferroni corrections (corrected for 3 comparisons).

Participants encountered velocity-dependent forces that displaced their arm lateral to the goal target. Figure 2A displays the hand paths of an exemplar participant. The representative participant made relatively straight unloaded movements in the baseline phase. Similar results were observed at the group level (Figure 2B). Peak lateral deviations in the baseline phase did not differ significantly across testing sessions (*F*_2, 81_ = 0.79, *p* = 0.46). Introducing the force field unexpectedly caused an abrupt and systematic increase in peak lateral deviations, which decayed systematically throughout adaptation until the participant’s movements were relatively accurate (Figure 2A). The participant made pronounced mirror hand-path deviations that decayed toward baseline performance when the forces were removed unexpectedly in the washout phase. Similar results were observed at the group level (Figure 2B).

Savings describes an improvement in adaptation when participants are re-exposed to the same force field (Smith et al., 2006; Coltman et al., 2019). We were interested in the amount of savings that participants express when they re-encounter the same force field. Linear mixed-effects models revealed a significant difference in peak lateral deviations in *Early Adaptation* (*F*_2, 81_ = 12.20, *p* < 0.001). *Post-hoc F*-tests revealed a pronounced reduction in the size of peak lateral deviations when participants were re-exposed to the forces in the 24-h (Initial and 24 h: *F*_1, 81_ = 16.69, *p* < 0.001; Figure 2C) and 1-week sessions (Initial and 1 week: *F*_1, 81_ = 19.77, *p* < 0.001; Figure 2C). Peak lateral deviations in *Early Adaptation* did not differ significantly across sessions at 24 h and 1 week (*F*_1, 81_ = 0.13, *p* = 0.72; Figure 2C). Peak lateral deviations in *Late Adaptation* did not differ significantly across sessions (*F*_2, 81_ = 1.88, *p* = 0.16; Figure 2D).

Aftereffects are hand-path deviations that mirror the direction of the force field when it is removed unexpectedly in the washout phase. They are often used as a proxy for the amount participants adapt their movements and actively compensate for the forces encountered during reaching (Shadmehr and Mussa-Ivaldi, 1994; Gandolfo et al., 1996; Conditt et al., 1997; Leow et al., 2018; Avraham et al., 2020). Linear mixed-effects models revealed a significant difference in peak lateral deviations in *Washout* (*F*_2, 81_ = 4.54, *p* = 0.01). *Post-hoc F*-tests showed a greater reduction in peak lateral deviations, on average, when the forces were removed unexpectedly in *Washout* when comparing the Initial session with the sessions at 24 h and 1 week (Initial and 24 h: *F*_1, 81_ = 6.35, *p* = 0.04; Initial and 1 week: *F*_1, 81_ = 7.25, *p* = 0.03; Figure 2E). Peak lateral deviations in *Washout* did not differ significantly between the sessions at 24 h and 1 week (*F*_1, 81_ = 0.03, *p* = 0.86). Taken together, the kinematic results reveal savings in *Early Adaptation* and a reduction in lateral deviations during *Washout* upon re-exposure to the same force field. On average, *Late Adaptation* did not differ significantly between exposures to the force field.

### Experiment 1: Individual Differences in Movement Kinematics Across Testing Sessions

**FIGURE 3:**
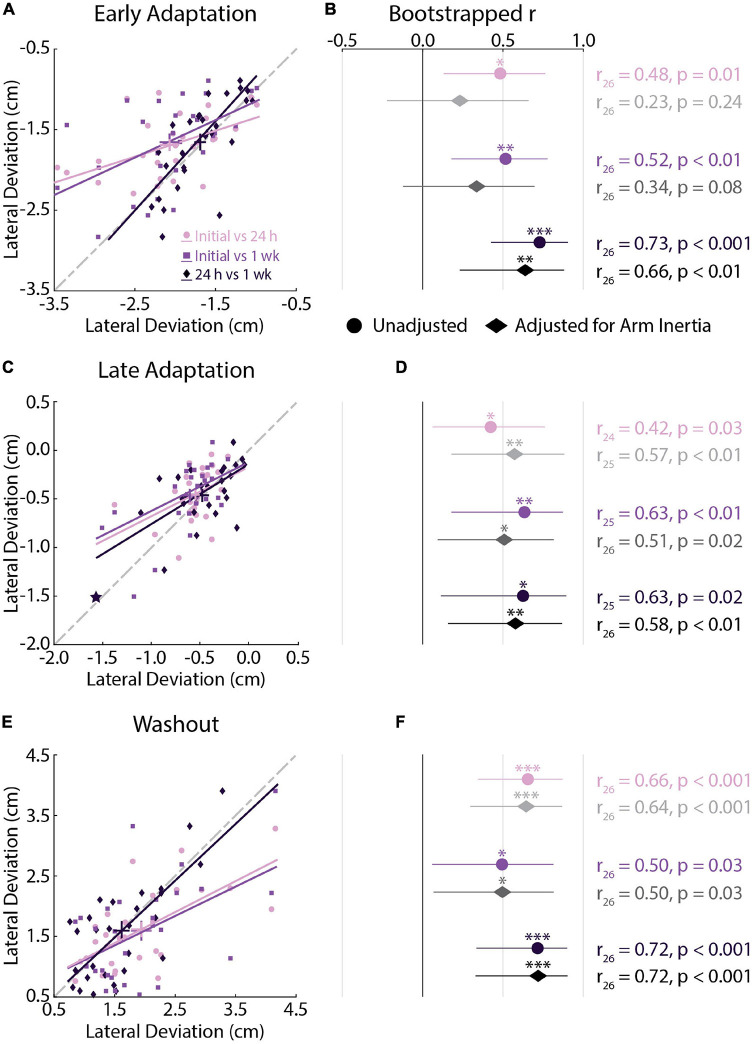
Kinematic correlations across testing sessions (*Experiment 1*). **(A)** Correlations between peak lateral deviations measured in *Early Adaptation* across all sessions. For each correlation, the earlier session is represented on the *x*-axis and later session on the *y*-axis. Bars represent the mean and SEM. Trendlines were obtained via robust linear regression and are used for visualization purposes. The dashed gray line represents unity. **(B)** Mean and 95% confidence intervals (CI) of the bootstrapped Pearson’s *r* distributions are shown for each correlation (circles) and for the partial Pearson’s correlations adjusted for arm inertia (diamonds). **(C,D)** Correlations between peak lateral deviations measured in *Late Adaptation* across all sessions. **(E,F)** Correlations between peak lateral deviations measured in *Washout* across all sessions. Data in panels **(C–F)** are presented in the same manner as panels **(A,B)**. **(C)** Star indicates the observation that did not reach the critical value for outlier detection. The correlation results were not different with and without this observation. *p < 0.05, **p < 0.01, ***p < 0.001 after Bonferroni corrections (corrected for 3 correlations).

We performed correlation analyses to examine the relationship between peak lateral deviations in each testing session. We used the analyses to examine if participants display reliable peak lateral deviations when repeatedly exposed to the same forces. The analyses revealed moderate-strong correlations in *Early Adaptation* (Figures 3A,B), *Late Adaptation* (Figures 3C,D), and *Washout* (Figures 3E,F), demonstrating that participants who displayed smaller kinematic errors in each phase of the experiment tended to do so in each testing session. We observed one visually distant data point in *Late Adaptation* that did not meet the threshold for outlier detection (star in Figure 3C). Removing this observation did not qualitatively change the results (*r*_24_ = 0.47, CI [0.04, 0.72], *p* = 0.049).

We repeated the analysis while accounting for the inertia of the arm. Past studies have shown that inertia can influence peak arm displacement when disturbed by force perturbations during posture control (Bourke et al., 2015). Partial correlations were performed to rule out differences in the inertia of the arm that may influence how participants seemingly adapt their movements. Accounting for the inertia of the arm reduced the strength of the relationship between peak lateral deviations in *Early Adaptation* (Figure 3B). The adjusted relationship was significant when examining the correlations between peak lateral deviations in the 24-h and 1-week sessions. There was little change in the strength of the correlations in *Late Adaptation* and *Washout* (Figures 3D,F). In short, participants who displayed smaller kinematic errors in each phase of the experiment tended to do so in each testing session.

We performed a difference in correlation analysis to examine changes in the strength of the relationship across repeated exposure to the force field (Table 1). The analysis did not reveal any significant differences in correlations across testing sessions. Qualitatively similar results were observed when examining correlations that accounted for the inertia of the arm. Collectively, the correlation analyses revealed reliable individual differences in peak lateral deviations that did not differ statistically across testing sessions.

**TABLE 1:** Differences in the strength of correlations between peak lateral deviations measured across testing sessions.

	Initial vs. 24 h—Initial vs. 1 week	Initial vs. 24 h—24 h vs. 1 week	Initial vs. 1 week—24 h vs. 1 week
**Early adaptation**			
Unadjusted	Δ*r* = –0.03, *p* = 0.69	Δ*r* = –0.25, *p* = 0.16	Δ*r* = –0.21, *p* = 0.21
Adjusted	Δ*r*_partial_ = –0.10, *p* = 0.69	Δ*r*_partial_ = –0.41, *p* = 0.10	Δ*r*_partial_ = –0.30, *p* = 0.19
**Late adaptation**			
Unadjusted	Δ*r* = –0.21, *p* = 0.26	Δ*r* = –0.20, *p* = 0.36	Δ*r* = –0.01, *p* = 0.93
Adjusted	Δ*r*_partial_ = 0.07, *p* = 0.79	Δ*r*_partial_ = –0.01, *p* = 0.97	Δ*r*_partial_ = –0.07, *p* = 0.75
**Washout**			
Unadjusted	Δ*r* = 0.16, *p* = 0.44	Δ*r* = –0.06, *p* = 0.67	Δ*r* = –0.22, *p* = 0.27
Adjusted	Δ*r*_partial_ = 0.15, *p* = 0.49	Δ*r*_partial_ = –0.08, *p* = 0.63	Δ*r*_partial_ = –0.22, *p* = 0.28

### Experiment 1: Average Differences in Force Compensation Across Testing Sessions

**FIGURE 4:**
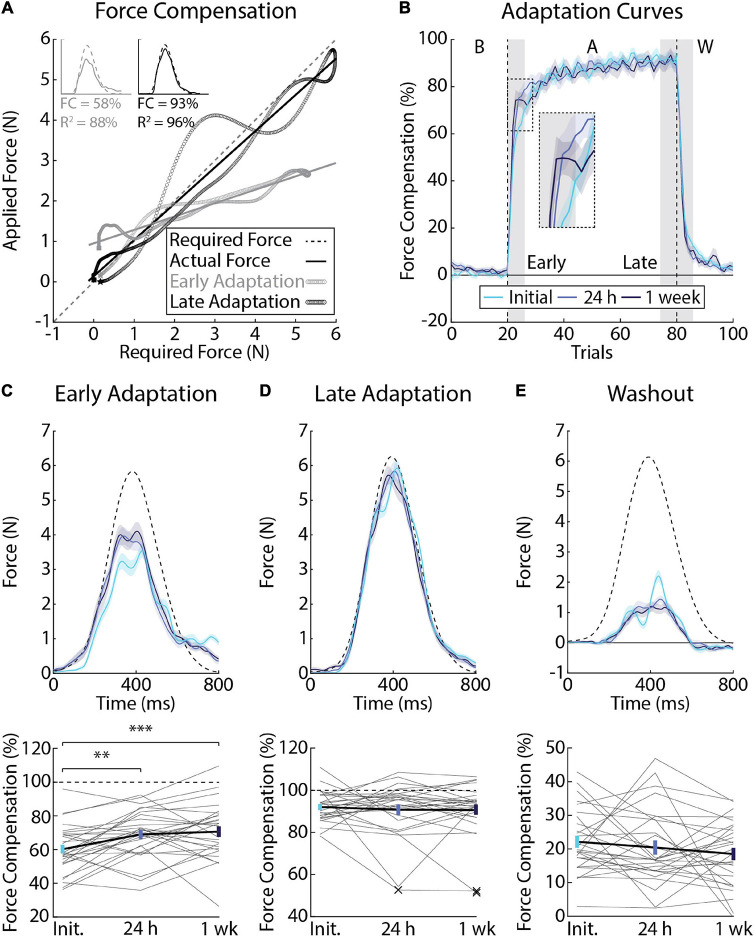
Differences in force compensation across testing sessions (*Experiment 1*). **(A)** Force profiles and linear regression from a representative participant showing the slope calculation for a single trial at the beginning (gray) and end (black) of adaptation. The force required to counter the force field is plotted on the *x*-axis and the actual force applied by the participant is plotted on the *y*-axis. Slopes were then converted to force compensation. **(B)** Average force compensation over the course of the experiment (*n* = 28, 12 female). Lines represent the means from each session and shaded regions the corresponding SEM. Individual differences in adaptation were measured in the first (*Early Adaptation*, inset panel) and last 3 blocks of adaptation (*Late Adaptation*) as well as the first 3 blocks of *Washout* (shaded gray regions). **(C)** Average force profiles and force compensation during *Early Adaptation*. Top: dashed line represents the ideal amount of force required to counter the force field, had it been applied. Solid lines represent mean force profiles and shaded regions indicate SEM. Bottom: gray lines indicate individual participant data measured across sessions. Error-bars indicate mean and SEM during *Early Adaptation*. Dashed line indicates force compensation = 100%. **(D)** Average force profiles and force compensation during *Late Adaptation*. “X” indicates multivariate outliers (Mahalanobis distance) that were excluded from the analysis. **(E)** Average force profiles and force compensation during *Washout*. Data in panels **(D,E)** are presented in the same manner as panel **(C)**. **p < 0.01 and ***p < 0.001 after Bonferroni corrections (corrected for 3 comparisons).

Error-clamps are often used to dissociate how participants adapt their planned movements from feedback corrections or non-specific mechanisms that can alter behavior (Orban De Xivry et al., 2011; Wu et al., 2014). In our experiment, participants performed 20 clamp trials in baseline, 60 during adaptation, and 20 in the washout phase. We quantified adaptation by regressing the forces that participants exerted on the channel walls with the forces required to compensate for the velocity-dependent force field, had it been applied (Figure 4A). As noted in previous studies (Joiner and Smith, 2008), force compensation approached 100% as participants learned to better approximate the force field in each session. Despite time periods where participants over- and undercompensated for the applied forces, the relationship between ideal and actual forces was generally well fit by linear regression (mean *R*^2^ = 83%, *SEM* = 6% across the adaptation phase; Figure 4A).

Figure 4B reveals savings, or an increase in the average amount of force compensation that participants displayed in *Early Adaptation* (*F*_2, 81_ = 8.42, *p* < 0.001). *Post-hoc F*-tests revealed an increase in force compensation when participants were re-exposed to the forces at 24 h (Initial and 24 h: *F*_1, 81_ = 10.36, *p* < 0.01) and 1 week later (Initial and 1 week: *F*_1,81_ = 14.54, *p* < 0.001; Figure 4C). Force compensation in *Early Adaptation* did not differ significantly between the 24-h and 1-week sessions (*F*_1, 81_ = 0.35, *p* = 0.55). Force compensation in *Late Adaptation* and *Washout* did not differ significantly across sessions (*Late Adaptation*: *F*_2, 78_ = 0.28, *p* = 0.75; Figure 4D; *Washout*: *F*_2, 81_ = 1.20, *p* = 0.31; Figure 4E).

### Experiment 1: Individual Differences in Force Compensation Across Testing Sessions

**FIGURE 5:**
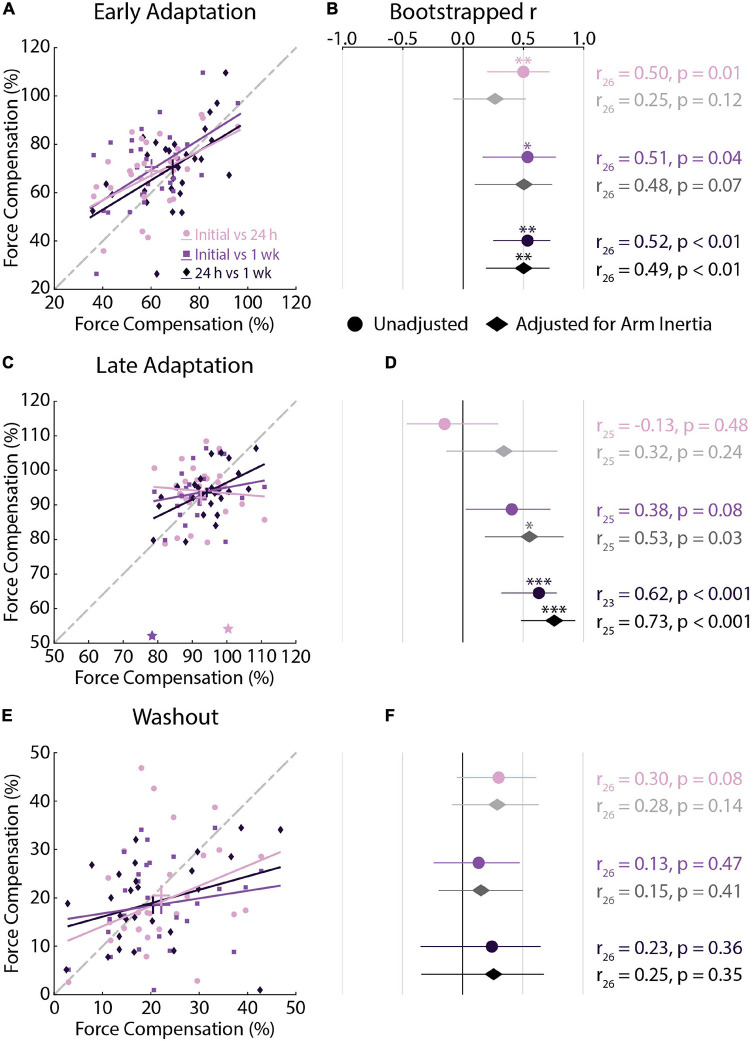
Force compensation correlations across testing sessions (*Experiment 1*). **(A)** Correlations between force compensation in *Early Adaptation* across all sessions. For each correlation, the earlier session is represented on the *x*-axis and later session on the *y*-axis. Bars represent the mean and SEM. Trendlines were obtained via robust linear regression and are used for visualization purposes. The dashed gray line represents unity. **(B)** Mean and 95% confidence intervals (CI) of the bootstrapped Pearson’s *r* distributions are shown for each correlation (circles) and for the partial Pearson’s correlations adjusted for arm inertia (diamonds). **(C,D)** Correlations between force compensation in *Late Adaptation* across all sessions. **(E,F)** Correlations between force compensation in *Washout* across all sessions. Data in panels **(C–F)** are presented in the same manner as panels **(A,B)**. **(C)** Stars indicate observations that did not reach the critical value for outlier detection. The results were not different with and without these observations. *p < 0.05, **p < 0.01, ***p < 0.001 after Bonferroni corrections (corrected for 3 correlations).

Force compensation in *Early Adaptation* was moderately correlated across all testing sessions (Figures 5A,B), whereas force compensation in *Late Adaptation* was strongly correlated between the 24-h and 1-week sessions (Figures 5C,D). In *Late Adaptation*, there were two visually distant observations that did not meet the threshold for outlier detection (Figure 5C). Removing these observations did not qualitatively alter the results (Initial vs. 24 h: *r*_24_ = 0.02, CI [–0.45, 0.52], *p* = 0.95; Initial vs. 1 week: *r*_24_ = 0.22, CI [–0.19, 0.60], *p* = 0.22). Overall, the results indicate that participants who better compensated for the forces in *Early* and *Late Adaptation* tended to do so across testing sessions. Force compensation in *Washout* was weakly correlated across all sessions (Figures 5E,F). Accounting for individual differences in the inertia of the arm led to qualitatively similar results (Figure 5). Finally, the difference in correlation analysis revealed only one difference between unadjusted relationships in *Late Adaptation* (Initial vs. 24 h and 24 h vs. 1 week; Table 2). Qualitatively similar results were observed for the adjusted correlations (Table 2). Taken together, the results show reliable individual differences in peak lateral deviations and force compensation with repeated exposure to a velocity-dependent force field.

**TABLE 2:** Differences in the strength of correlations between force compensation across testing sessions.

	Initial vs. 24 h—Initial vs. 1 week	Initial vs. 24 h—24 h vs. 1 week	Initial vs. 1 week—24 h vs. 1 week
**Early adaptation**			
Unadjusted	Δ*r* = –0.02, *p* = 0.91	Δ*r* = –0.03, *p* = 0.89	Δ*r* = –0.01, *p* = 0.99
Adjusted	Δ*r*_partial_ = –0.23, *p* = 0.29	Δ*r*_partial_ = –0.24, *p* = 0.23	Δ*r*_partial_ = –0.01, *p* = 0.99
**Late adaptation**			
Unadjusted	Δ*r* = –0.50, *p* = 0.09	Δ*r* = –0.74, *p* = 0.01*	Δ*r* = –0.24, *p* = 0.24
Adjusted	Δ*r*_partial_ = –0.21, *p* = 0.49	Δ*r*_partial_ = –0.40, *p* = 0.12	Δ*r*_partial_ = –0.20, *p* = 0.34
**Washout**			
Unadjusted	Δ*r* = 0.17, *p* = 0.50	Δ*r* = 0.07, *p* = 0.85	Δ*r* = –0.10, *p* = 0.73
Adjusted	Δ*r*_partial_ = 0.14, *p* = 0.60	Δ*r*_partial_ = 0.04, *p* = 0.93	Δ*r*_partial_ = –0.10, *p* = 0.74

### Experiment 2a: Velocity- and Position-Dependent Forces

**FIGURE 6:**
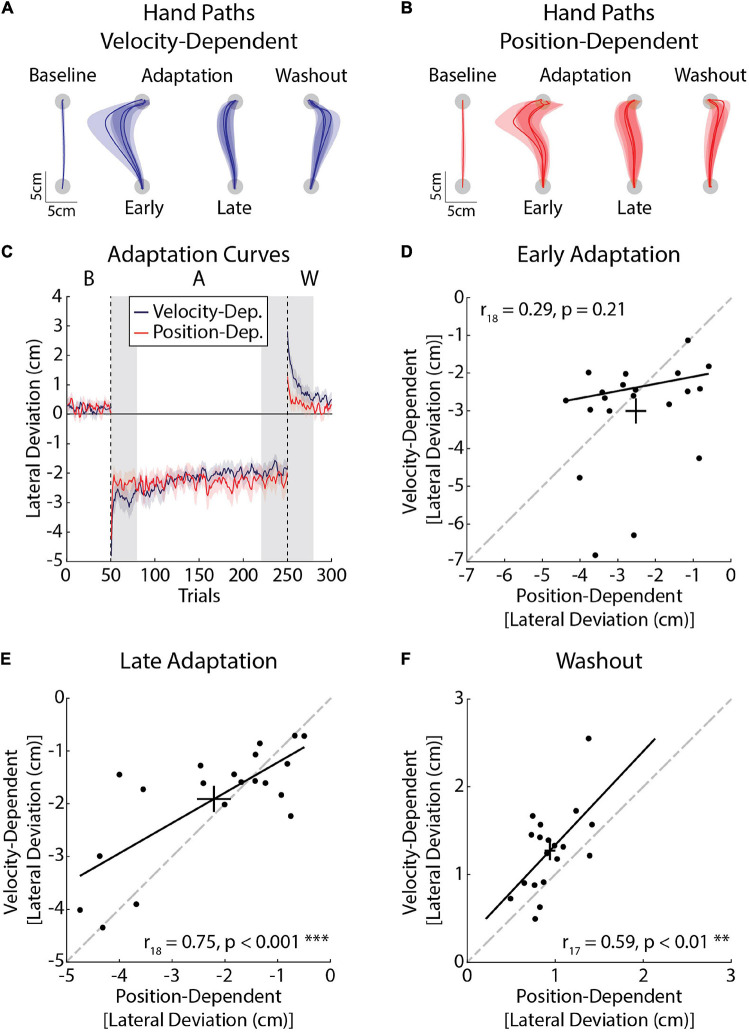
Kinematic differences in adaptation in velocity- (blue) and position-dependent (red) force fields (*Experiment 2a*). **(A)** Average hand paths from the velocity-dependent force field. Hand paths were averaged across the baseline phase. The first and last three trials of the adaptation phase were averaged across participants. Similarly the first three trials of the washout phase were averaged across participants. **(B)** Average hand paths from the position-dependent force field. Hand paths are presented in the same manner as panel **(A)**. **(C)** Peak lateral deviations throughout the experiment (*n* = 20, 10 female). Solid lines represent the group mean and surrounding shaded regions represent the SEM. Individual differences in adaptation were measured in *Early Adaptation*, *Late Adaptation*, and *Washout* (shaded gray regions). **(D)** Average peak lateral deviations measured in *Early Adaptation* in each task. Peak lateral deviations for the position-dependent forces are plotted on the *x*-axis and peak lateral deviations for the velocity-dependent forces are plotted on the *y*-axis. Error bars represent the mean and SEM. The trendline was obtained via robust linear regression and is used for visualization purposes. The dashed line represents unity. **(E)** Average peak lateral deviations measured in *Late Adaptation*. **(F)** Average peak lateral deviations measured in *Washout*. Results in panels **(E,F)** are presented in the same format as panel **(D)**. **p < 0.01 and ***p < 0.001 after Bonferroni corrections (corrected for 3 comparisons).

Our first experiment revealed reliable individual differences in peak lateral deviations and force compensation when participants adapted to the same force field. Here, we examined how individual participants adapted to different force environments. Within a single session, participants reached in environments where their arm was disturbed by lateral forces that were proportional to their forward reaching velocity or position of their hand relative to the start and goal targets (Figures 6A,B). Baseline deviations did not differ significantly between tasks (*t*_19_ = 0.36, *p* = 0.72). We did not observe any differences in peak lateral deviations in *Early Adaptation* (*t*_19_ = 1.36, *p* = 0.19; Figure 6C), nor did the deviations in *Early Adaptation* correlate between the tasks (*r*_18_ = 0.29, CI [–0.14, 0.59], *p* = 0.21; Figure 6D). The extent that participants adapted their movements by *Late Adaptation* was not significantly different (*t*_19_ = –1.41, *p* = 0.17), but there was a strong relationship across tasks (*r*_18_ = 0.75, CI [0.37, 0.90], *p* < 0.001; Figure 6E). Peak lateral deviations in *Washout* (aftereffects) were larger in the velocity-dependent task (*t*_19_ = –3.79, *p* < 0.01) and correlated across the tasks (*r*_17_ = 0.59, CI [0.22, 0.82], *p* < 0.01; Figure 6F). Accounting for the inertia of the arm did not alter the relationship between *Early Adaptation*, *Late Adaptation*, or *Washout* across tasks (*Early Adaptation*: *r*_18_ = 0.28, CI [–0.21, 0.68], *p* = 0.17; *Late Adaptation*: *r*_18_ = 0.72, CI [0.31, 0.94], *p* < 0.01; *Washout*: *r*_17_ = 0.56, CI [0.13, 0.84], *p* = 0.01). Thus, participants who displayed more pronounced reductions in peak lateral deviations and larger aftereffects did so when countering velocity- and position-dependent forces.

### Experiment 2b: Velocity-Dependent Forces and Visuomotor Rotation

**FIGURE 7:**
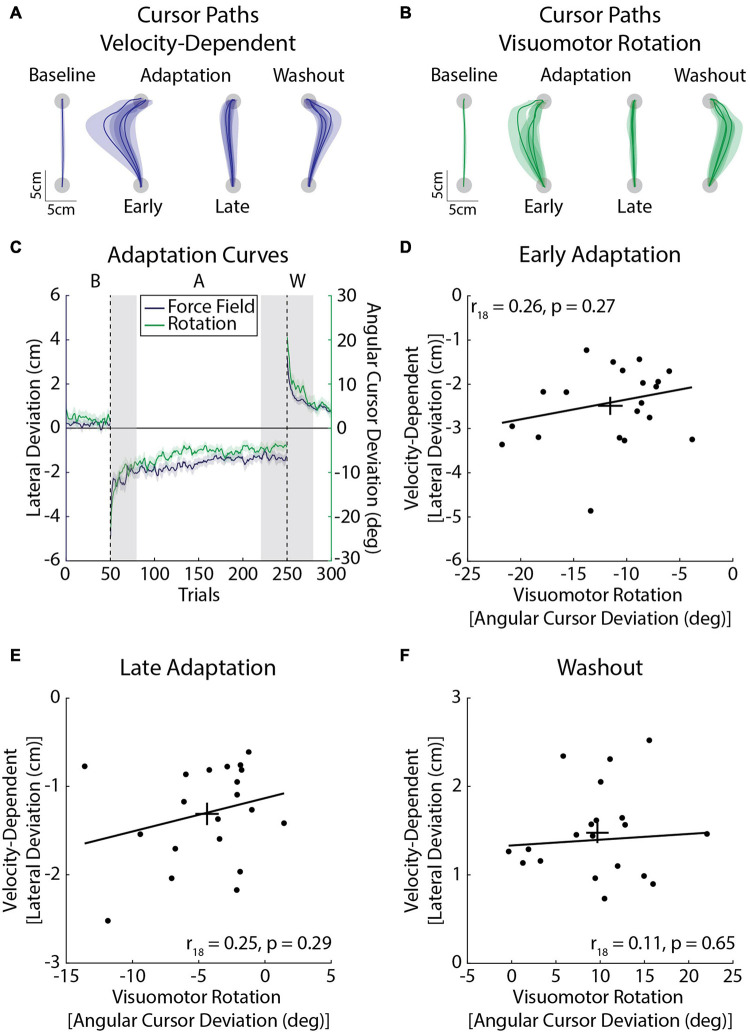
Average adaptation for the velocity-dependent force-field (blue) and visuomotor rotation (green) task (*Experiment 2b*; *n* = 20, 10 female). **(A)** Average cursor paths from the velocity-dependent force field. Cursor paths were averaged across the baseline phase. The first and last three trials of the adaptation phase were averaged across participants. Similarly the first three trials of the washout phase were averaged across participants. Note that cursor and hand paths were the same in the velocity-dependent force field. **(B)** Average cursor paths from the visuomotor rotation. Cursor paths are presented in the same manner as panel **(A)**. **(C)** Peak lateral and angular cursor deviations throughout the experiment. The line represents the group mean and the shaded regions represent the SEM. Individual differences in adaptation were measured in *Early Adaptation*, *Late Adaptation*, and *Washout* (shaded gray regions). The left *y*-axis corresponds to the velocity-dependent force-field task (peak lateral deviations) and the right *y*-axis to the visuomotor rotation task (cursor angle). **(D)** Amount of adaptation in *Early Adaptation* in the force field and rotation tasks. The angular deviations of the cursor in the visuomotor rotation task are plotted on the *x*-axis and peak lateral deviations for the velocity-dependent force task are plotted on the *y*-axis. The trendline was obtained via robust linear regression and is used for visualization purposes. Error bars represent the mean and SEM. **(E)** Average amount of adaptation in *Late Adaptation.* **(F)** Average peak lateral and angular cursor deviations in *Washout.* Data in panels **(E,F)** are presented in the same format as panel **(D)**.

In this experiment, participants adapted to a velocity-dependent force field and visuomotor rotation during a single session of reaching movements (Figures 7A–C). We did not find any significant correlations between the amount of adaptation expressed in *Early Adaptation* (*r*_18_ = 0.26, CI [–0.18, 0.55], *p* = 0.27; Figure 7D), *Late Adaptation*: *r*_18_ = 0.25, CI [–0.28, 0.73], *p* = 0.29; Figure 7E), or *Washout* (*r*_18_ = 0.11, CI [–0.26, 0.49], *p* = 0.65; Figure 7F). Accounting for the inertia of the arm had little effect on the results (*Early Adaptation*: *r*_18_ = 0.41, CI [–0.08, 0.74], *p* = 0.08; *Late Adaptation*: *r*_18_ = 0.21, CI [–0.33, 0.74], *p* = 0.37; *Washout*: *r*_18_ = 0.08, CI [–0.42, 0.56], *p* = 0.66).

## Discussion

We examined how healthy young adults adapted their reaching movements when interacting with force fields and a visuomotor rotation. In our first experiment, we found that participants displayed moderate-strong relationships in movement kinematics (peak lateral deviations) and weak-strong relationships in force compensation when they adapted to the same force field in three sessions. Our second experiment investigated individual differences in adaptation when exposed to position- and velocity-dependent forces, or velocity-dependent forces and a visuomotor rotation. Participants displayed moderate-strong correlations in *Late Adaptation* and *Washout* when exposed to position- and velocity-dependent forces but weak relationships when adapting to velocity-dependent forces and a visuomotor rotation. Collectively, the experiments characterize individual differences in adaptation across broad but common sensorimotor disturbances.

### Average Adaptation With Repeated Force-Field Exposure

Savings describes an improvement in adaptation when participants are re-exposed to the same force field (Smith et al., 2006; Coltman et al., 2019). Consistent with a number of studies, we observed improvements in the average amount of *Early Adaptation* when participants re-encountered the force field at 24 h after initial exposure. *Early Adaptation* was still improved after 1 week without practice. Similar results were observed in peak lateral deviations in force field trials and force compensation in error clamp trials. Past studies have reported savings within a single session, often separated by washout (Yin and Wei, 2020), or counter-adaptation trials (Coltman et al., 2019). Other studies have demonstrated that savings between consecutive days may depend on the length of initial exposure and presence of washout trials between sessions (Nguyen et al., 2019). Our findings show that savings is present across sessions separated by washout trials and is conserved for up to a week without practice. The results do not preclude long-term savings that have been observed with extended visuomotor adaptation (Landi et al., 2011).

We also assessed peak lateral deviations and the extent of force compensation that participants displayed in *Late Adaptation.* There were no differences in either measure in *Late Adaptation* when participants were re-exposed to the force field. This finding is consistent with evidence that the overall extent of adaptation tends to stabilize within the first session of 4 weeks of force-field adaptation (Nezafat et al., 2001), and does not differ, on average, after 24 h to 1 week without practice (Caithness et al., 2004). Past studies have reported similar average amounts of force compensation when participants are re-exposed to a velocity-dependent force field in a single testing session (Coltman et al., 2019). We extend these findings by showing that average amounts of force compensation in *Late Adaptation* lack significant differences across sessions spanning over a week.

Aftereffects have been used to quantify the degree to which participants adapt their reaching movements to compensate for the effects of the force field (Shadmehr and Mussa-Ivaldi, 1994; Gandolfo et al., 1996; Conditt et al., 1997). A lack of aftereffects has been associated with a reduction in adaptation (Maschke et al., 2004; Smith and Shadmehr, 2005; Donchin et al., 2011). We found that kinematic aftereffects were reduced when re-assessed at 24 h after initial exposure and 1 week after the 24-h session. This observation is consistent with research showing reduced aftereffects when participants were re-exposed to a visuomotor rotation (Avraham et al., 2020). The results showed no significant differences in force compensation during *Washout.*

### Individual Differences in Adaptation Are Reliable Across Testing Sessions

Force-field adaptation has primarily been studied through the lens of group averages. While this has broadened our understanding of factors that influence adaptation, the approach de-emphasizes differences in how individuals adapt their movements. Here we focused on individual differences in force-field adaptation. We investigated the relationship between peak lateral deviations in force-field trials as well as force compensation expressed in clamp trials across repeated testing. We found that participants tend to display correlated peak lateral deviations and force compensation in *Early* and *Late Adaptation*. Our results compliment recent studies that characterized individual differences in visuomotor adaptation (Stark-Inbar et al., 2017; Oh and Schweighofer, 2019; Tsay et al., 2021; Wilterson and Taylor, 2021). We build upon these studies and provide a foundation for future work examining factors that influence individual differences in force-field adaptation.

Humans can vary widely in their physical attributes. Individual differences in body morphology alter the inertia of the arm, and accordingly, motion when disturbed by a physical perturbation. Given that larger hand deviations can increase adaptation (Thoroughman and Shadmehr, 2000; Scheidt et al., 2001; Joiner and Smith, 2008; Wei and Körding, 2010; Patton et al., 2013), we wanted to rule out the simple explanation that individual differences in adaptation arise from variation in the inertia of the arm. Individual differences in *Early* and *Late Adaptation* were correlated across sessions, both in terms of peak lateral deviations in force-field trials and force compensation in error-clamp trials. Importantly, individual differences in adaptation were not explained by variation in the physical attributes of our participants.

There are several factors that may contribute to individual differences in adaptation. One possible explanation is that individual differences stem from the memory of a reinforced pattern of movement (Orban De Xivry and Lefèvre, 2015), such that participants repeat behaviors that led to success in the initial testing session. Another possible explanation is that individual differences in adaptation reflect the ability to recall an implicit motor memory of adaptation (Thoroughman and Shadmehr, 1999; Takiyama et al., 2015) possibly triggered by the history of errors upon re-exposure to the force field (Herzfeld et al., 2014b; but see Coltman et al., 2021). Consistent with this idea, past studies have shown that day-to-day changes in behavior may be linked to the implicit component of adaptation (Joiner and Smith, 2008; Takiyama et al., 2015; Yin and Wei, 2020), while others have revealed individual differences in implicit visuomotor adaptation that are reliable across testing sessions (Stark-Inbar et al., 2017; Wilterson and Taylor, 2021).

Other studies caution that it may be difficult to attribute adaptation solely to motor memories (Huberdeau et al., 2015; Morehead et al., 2015; Leow et al., 2020). Avraham et al. (2020) used clamped visual feedback and explicit instruction to dissociate contributions from implicit and explicit processes in visuomotor adaptation. Although the average amount of adaptation was similar upon re-exposure to the visuomotor rotation, the authors found that explicit strategies accounted for a greater proportion of adaptation, both in early re-exposure and long after adaptation had reached an asymptote. Thus, the relative contributions of explicit and implicit processes may evolve in the absence of quantifiable changes in movement kinematics (Avraham et al., 2020). Similar mechanisms may be at play in our experiment, where participants reduced kinematic deviations to produce accurate reaching movements. Studies that attempt to dissociate explicit and implicit processes in force-field adaptation would help in characterizing whether these processes change with repeated exposure to a force field (Schween et al., 2020).

We found conflicting results when examining kinematic and clamp-based measures of *Washout*. While participants displayed reliable peak lateral deviations in *Washout*, there were weak relationships between force compensation measured in the clamp across sessions. This discrepancy may reveal a limitation in interleaving null and clamp trials in the washout phase. The null trials resulted in pronounced aftereffects, which may have caused force compensation to return more rapidly to baseline performance when compared with studies that clamped hand paths and measured de-adaptation in the absence of salient movement errors (Scheidt et al., 2000; Pekny et al., 2011). It is possible that the presence of aftereffects caused mixed results in *Washout*. This may explain why we found that kinematic aftereffects decreased across sessions in the absence of comparable changes in average amounts of force compensation, and only weak correlations in force compensation in *Washout* across sessions. Another possible limitation is that single target adaptation may favor the use of explicit strategies (Bond and Taylor, 2015). Although single target paradigms favor explicit strategies in visuomotor adaptation (Bond and Taylor, 2015), it is unclear whether a similar influence exists for force-field adaptation (Forano et al., 2021).

### Individual Differences in Adaptation Present When Adapting to Different Force Fields

In our second experiment, we observed a strong relationship in the amount participants adapted to the different forces in *Late Adaptation*, as well as in the amplitude of the aftereffects they displayed in *Washout*. The correlations occurred despite differences in amplitude and location of peak forces in the position- and velocity-based force fields, and a lack of correlation between tasks (Supplementary Material). Thus, participants appeared to adapt differently in *Early Adaptation*, but individual differences in adaptation became evident in *Late Adaptation* and *Washout.* Previous work suggests adaptation relies on a set of motor primitives that encode limb position and velocity, perhaps arising from the sensitivity of motor cortical neurons and peripheral receptors to changes in the state of the limb (Sing et al., 2009). Interference between opposing position- and velocity-dependent force fields supports this idea, as it would impact the tuning of primitives when exposed to the opposing force field (Bays et al., 2005; Sing et al., 2009). Importantly, the representation of position- and velocity-based forces showed a degree of overlap, despite differences in the location and timing of peak forces across tasks (Sing et al., 2009). In agreement with past work, the correlations in *Late Adaptation* and *Washout* reported here suggest the representation of the novel dynamics may be similar across force-field tasks (Sing et al., 2009).

We found weak relationships between measures of force-field and visuomotor adaptation. The partial correlations showed that individual differences in limb inertia had little influence on any relationship between force-field and visuomotor adaptation. Similar results were observed when the analysis was performed using peak lateral deviations (Supplementary Material). Thus, there may be differences in behavioral processes involved in adapting to force fields and visuomotor rotations (Krakauer et al., 1999). Other evidence suggests that force-field and visuomotor adaptation rely on distinct brain networks (Seidler, 2010). The cerebellum has been implicated in force-field (Maschke et al., 2004; Diedrichsen, 2005; Bastian, 2006; Ostry et al., 2010; Taylor et al., 2014) and visuomotor adaptation (Diedrichsen, 2005; Graydon et al., 2005; Della-Maggiore et al., 2009). However, the posterior cerebellum may be more involved in visuomotor adaptation and the anterior cerebellum in adapting to force fields (Rabe et al., 2009; Donchin et al., 2011). A potential limitation of our experiment was the single-target design which may have led to more rapid adaptation (Bond and Taylor, 2015; Forano et al., 2021) and the possibility for ceiling effects that would weaken the correlations between force-field and visuomotor adaptation. However, Figures 7D,E show variability even when participants approached full adaptation, suggesting that ceiling effects alone could not explain the weak correlations between adaptation in the force-field and visuomotor rotation tasks. Taken together, our results show that reliable individual differences are present in force-field adaptation across multiple testing sessions and when participants adapt their movements to different force fields.

### Summary

We show that participants adapt their reaching movements in a reliable manner when repeatedly exposed to the same force field. Individual differences were evident when participants adapted their movements to different force fields, but weakly related between force-field and visuomotor rotation tasks. The approach is by definition correlative, but provides an understanding of individual differences in sensorimotor adaptation. Addressing this knowledge gap will be an essential step for understanding motor learning deficits that arise in clinical populations, how these impairments might change with disease progression, or improve over the course of neurorehabilitation.

## Data Availability Statement

The raw data supporting the conclusions of this article will be made available by the authors, without undue reservation.

## Ethics Statement

The studies involving human participants were reviewed and approved by the Conjoint Health Research Ethics Board at the University of Calgary. The patients/participants provided their written informed consent to participate in this study.

## Author Contributions

RM and TC contributed to the design of the study, performed the data analysis, and prepared the final manuscript. RM collected the data and composed the initial draft of the manuscript. Both authors contributed to the article and approved the submitted version.

## Conflict of Interest

The authors declare that the research was conducted in the absence of any commercial or financial relationships that could be construed as a potential conflict of interest.

## Publisher’s Note

All claims expressed in this article are solely those of the authors and do not necessarily represent those of their affiliated organizations, or those of the publisher, the editors and the reviewers. Any product that may be evaluated in this article, or claim that may be made by its manufacturer, is not guaranteed or endorsed by the publisher.
