# Supplementary material for: Individual Differences in Sensorimotor Adaptation Are Conserved Over Time and Across Force-Field Tasks
Source: Front Hum Neurosci. 2021 Nov 30;15:692181. doi: 10.3389/fnhum.2021.692181 (PMC8669441; doi:10.3389/fnhum.2021.692181)
Supplement: Supplementary file 1 [file Data_Sheet_1.PDF]

Supplementary Material

1 Experiment 2a: Velocity- and Position-Dependent Forces

We performed supplementary analyses to compare the position- and velocity-dependent forces that participants encountered in *Experiment 2a*. We compared the amplitude and location of peak forces as well as the location of peak lateral deviations across tasks. Separate analyses were performed for *Early* and *Late Adaptation*.

Peak Force and Location of Peak Force

Early Adaptation: Peak forces were smaller in the position- (mean = 4.68, SEM = 0.002, range = 4.67 - 4.71 N) compared to velocity-dependent field (mean = 5.40, SEM = 0.25, range = 3.44 - 8.13 N; t_{19} = -2.91, p = 0.01). The peak forces also occurred later in the position- (mean = 7.51, SEM = 0.001, range = 7.50 - 7.52 cm; relative to the start target) compared to velocity-dependent field (mean = 6.93, SEM = 0.17, range = 5.82 - 10.83 cm; t_{19} = 3.32, p = 0.004). There were no correlations between peak forces (r_{18} = -0.08, CI [-0.50, 0.72], p = 0.62) or the location of the peak forces across tasks (r_{18} = -0.01, CI [-0.23, 0.53], p = 0.96; Supp. Figure 1).

Late Adaptation: Peak forces were smaller in the position- (mean = 4.67, SEM = 0.002, range = 4.66-4.70 N) compared to velocity-dependent field (mean = 5.56, SEM = 0.27, range = 3.77 - 8.27 N; t_{19} = -3.32, p = 0.004). Peak forces occurred farther into the reach in the position- (mean = 7.51, SEM = 0.001, range = 7.50 - 7.52 cm) compared to velocity-dependent field (mean = 7.11, SEM = 0.15, range = 5.67 - 8.20 cm; t_{19} = 2.74, p = 0.01). There were no correlations between peak force (r_{18} = -0.09, CI [-0.43, 0.40], p = 0.71) or the location of peak forces across tasks (r_{18} = -0.07, CI [-0.43, 0.30], p = 0.77; Supp. Figure 1).

Location of Peak Lateral Deviations

Early Adaptation: There was a weak correlation in the location of peak lateral deviations across tasks (r_{18} = 0.37, CI [-0.05, 0.70], p = 0.11). Accounting for the location of the peak errors did not appreciably change the relationship between peak lateral deviations presented in the main text ($r_{partial}$ = 0.27, CI [-0.22, 0.63], p = 0.26; Figure 6D).

Late Adaptation: There was a moderate correlation in the location of peak lateral deviations across tasks (r_{18} = 0.46, CI [0.05, 0.78], p = 0.04). Partial correlations accounting for this relationship did not alter the individual differences reported in text ($r_{partial}$ = 0.70, CI [0.29, 0.89], p < 0.001; Figure 6E).

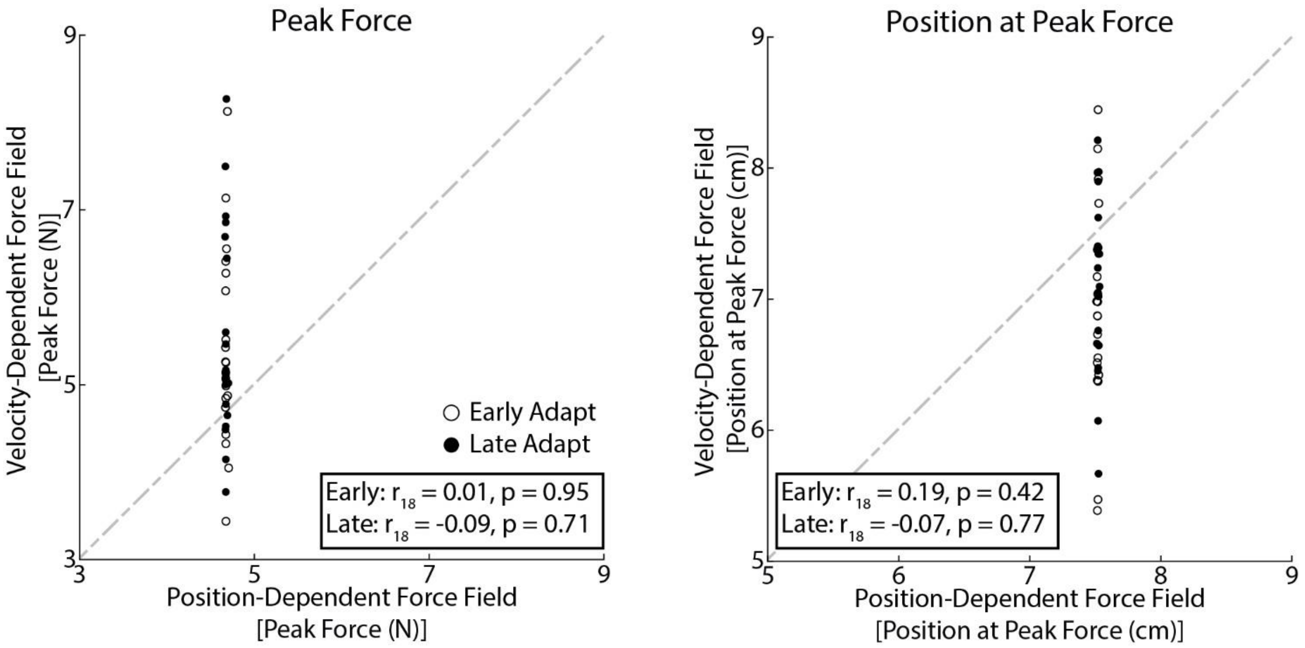

Supplementary Figure 1. Peak force and the location at which peak force was produced in the position- (x-axis) and velocity-dependent force fields (y-axis). Dashed grey line is unity.

2 Experiment 2b: Velocity-Dependent Forces and Visuomotor Rotation

We performed supplementary analyses to compare the amplitude and location of peak lateral deviations across the velocity-dependent forces and visuomotor rotation tasks in *Experiment 2b*. The amplitude and location of peak lateral deviations were calculated from cursor displacements (Supplementary Figure 2). Separate analyses were performed for *Early* and *Late Adaptation*.

Peak Lateral Deviations

Early Adaptation: Peak lateral deviations were smaller in the visuomotor rotation task (mean = -1.80, SEM = 0.16, range = -3.67 - -0.81 cm) compared to the force field (mean = -2.49, SEM = 0.20, range = -4.86 - -1.23 cm; t_{19} = 2.54, p = 0.02). Peak lateral deviations occurred earlier in the visuomotor rotation task (mean = 8.91, SEM = 0.28, range = 5.96 – 11.35 cm) compared to the force field (mean = 11.48, SEM = 0.25, range = 8.66 – 13.40 cm; t_{19} = 7.73, p < 0.001). Repeating the correlation analysis using peak lateral deviations did not change the results appreciably when compared to the analysis presented in the main text (r_{18} = 0.08, CI [-0.28, 0.32], p = 0.55; Figure 7D). The location of peak lateral deviations did not correlate across tasks (r_{18} = 0.19, CI [-0.21, 0.48], p = 0.27).

Late Adaptation: Peak lateral deviations were smaller in the visuomotor rotation task (mean = -0.61, SEM = 0.12, range = -2.41 - -0.10 cm) compared to the force field (mean = -1.31, SEM = 0.12,

range = -2.52 - -0.61 cm; t_{19} = 3.15, $p < 0.01$). Peak lateral deviations occurred earlier in the visuomotor rotation task (mean = 9.19, SEM = 0.31, range = 5.19 – 10.82 cm) compared to the force field (mean = 10.85, SEM = 0.22, range = 8.63 – 12.90 cm; t_{19} = 4.41, $p < 0.001$). Repeating the correlation analysis using peak lateral deviations produced qualitatively similar results as presented in the text (r_{18} = -0.09, CI [-0.43, 0.22], p = 0.56; Figure 7E). The location of peak lateral deviations did not correlate across tasks (r_{18} = 0.02, CI [-0.31, 0.39], p = 0.88).

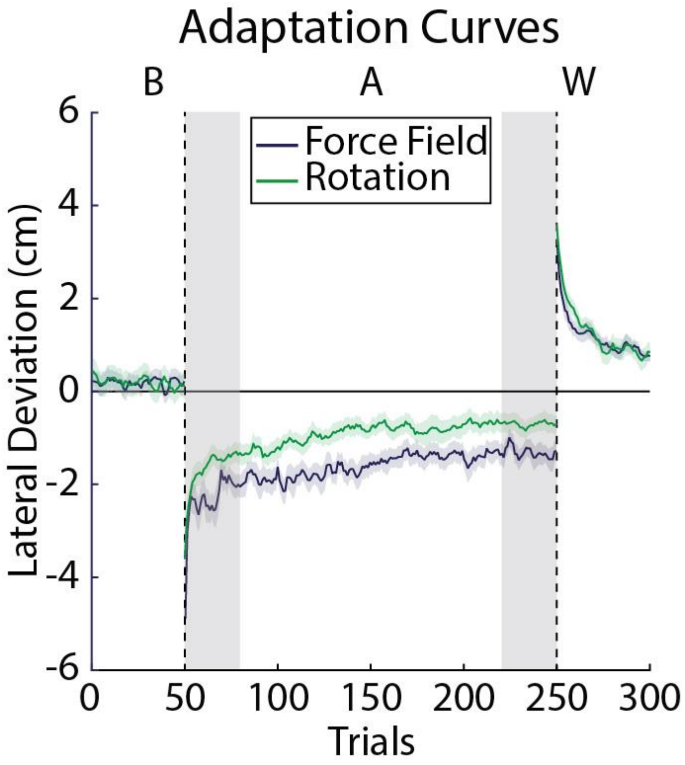

Supplementary Figure 2. Average peak lateral deviations for the velocity-dependent force-field (blue) and visuomotor rotation (green) task (*Experiment 2b*). The line represents the group mean and the shaded curve traces represent the SEM. Individual differences in adaptation were measured in *Early Adaptation* and *Late Adaptation* (shaded grey regions).
